# Caregiver Depression and Early Child Development: A Mixed-Methods Study From Rural China

**DOI:** 10.3389/fpsyg.2018.02500

**Published:** 2018-12-10

**Authors:** Ai Yue, Jiaqi Gao, Meredith Yang, Lena Swinnen, Alexis Medina, Scott Rozelle

**Affiliations:** ^1^Center for Experimental Economics in Education (CEEE), Shaanxi Normal University, Xi'an, China; ^2^Rural Education Action Program (REAP), Freeman Spogli Institute for International Studies, Stanford University, Stanford, CA, United States

**Keywords:** depression, rural China, female caregivers, child development, qualitative study, mixed methods analysis

## Abstract

Half of rural toddlers aged 0–3 years in China's Qinling Mountainous region are cognitively delayed. While recent studies have linked poor child development measures to the absence of positive parenting behaviors, much less is known about the role that caregiver depression might play in shaping child development. In this paper, a mixed methods analysis is used to explore the prevalence of depression; measure the association between caregiver depression and children's developmental delays, correlates of depression, and the potential reasons for caregiver depression among women in rural China. The analysis brings together results from a large-scale survey of 1,787 caregivers across 118 villages in one northwestern province, as well as information from in-depth interviews with 55 female caregivers from these same study sites. Participants were asked to respond to the Depression, Anxiety and Stress Scale-21 (DASS-21) as well as a scale to measure children's social-emotional development, the Ages and Stages Questionnaire: Social-Emotional (ASQ-SE). We also administered a test of early childhood development, the Bayley Scales of Infant and Toddler Development (BSID-III), to all of the study household's infants and toddlers. The results show that the prevalence of depression may be as high as 23.5 percent among all female caregivers (defined as scoring in the mild or higher category of the DASS-21). Grandmothers have higher prevalence of depression than mother caregivers (*p* < 0.01). Caregiver depression also is significantly associated with a 0.53 SD worsening of children's social-emotional development (*p* < 0.01) and a 0.12 SD decrease in children's language development (*p* < 0.05). Our qualitative findings reveal six predominant reasons for caregiver depression: lack of social support from family and friends; the burden of caregiving; lack of control and agency within the household; within-family conflict; poverty; the perception of material wealth as a measure of self-worth. Our findings show a serious lack of understanding of mental health issues among rural women, and suggest that rural communities could benefit greatly from an educational program concerning mental health and its influence on child development. Our findings confirm the need for a comprehensive approach toward rural health, with particular attention paid to mental health awareness and support to elderly caregivers.

## Introduction

Half of rural toddlers under the age of three in China's Qinling Mountain Region are cognitively delayed (Yue et al., 2017). It is commonly understood that such early delays are long-lasting and have implications for adult outcomes (Knudsen et al., 2006; Currie and Almond, 2011; Attanasio et al., 2015). Not only is this statistic alarming, it also demonstrates the need to better understand why these delays are occurring.

Recent studies have linked poor cognitive development in rural China to an absence of positive parenting practices (Wei et al., 2015; Yue et al., 2017). In one of the only large-scale empirical studies of parenting environments in rural China (Yue et al., 2017), it was found that a high fraction of caregivers (almost exclusively mothers and grandmothers) living in low-income areas of rural China do not regularly engage in positive parenting practices, such as reading to or singing to their children. The majority of caregivers (87.4 and 62.5%, respectively) did not read or sing to their children on the day prior to survey administration. More than half of families do not own any children's books (Yue et al., 2017). These findings are suggestive of the limited parent-child interaction in rural Chinese households.

While parenting behavior is influenced by many factors, one factor that has been shown to be important is caregiver depression. Depression is common among mothers with young children, with global prevalence levels ranging from 13 to 21% (O'Hara and Swain, 1996; Coll et al., 2016; de Castro et al., 2017). Numerous studies have found that the depressive states of caregivers can have adverse effects on parenting (Whiffen and Gotlib, 1989; Downey and Coyne, 1990; Lovejoy et al., 2000; Cummings and Kouros, 2009; Goodman et al., 2011). One study in the UK found that if the mother scored in the top 15% of a normal population for depressive symptoms, her child's risk for a mental disorder at age 13 were doubled (O'Donnell et al., 2014). A meta-analysis conducted by Lovejoy et al. (2000) found that the long-term effects of caregiver depression are especially important for infants, toddlers and young children, as depressed mothers display more negative interactions with their children across domains that are directly relevant for children's development. Research in developing countries has also consistently found that young children of depressed mothers have lower levels of cognitive function (Patel et al., 2003; Black et al., 2007).

There are many different mechanisms by which maternal depression can negatively affect child outcomes. Children may learn from their parents through observation and exposure to their depressive behaviors, such as withdrawing rather than interacting in interpersonal challenges (Downey and Coyne, 1990). In fact, the same study also found that depressed mothers are more likely to interact with their children in the same ways in which depressed people have been shown to interact with other adults: with limited facial and behavioral affect, with hostility, or with a tendency toward negative affect. Subsequent research has shown that depressed mothers tend to be more disengaged, irritable, and hostile toward their children and less likely to engage in stimulating activities during their child's sensitive developmental period (Lovejoy et al., 2000; Pelaez et al., 2008).

There is reason to believe that depression might be a serious problem in rural China. A nation-wide study of 3,824 elderly (aged 60 or older) in rural China using the Center for Epidemiologic Studies Depression Scale (CES-D) found that 38.7% of participants were depressed (Li et al., 2015). Another study found that the prevalence of depression among rural women in Sichuan was 12.4% (Hou et al., 2015; Qiu et al., 2016). While we know of no national data on depression in rural areas among caregivers specifically, smaller, regionally-focused studies have indicated a high prevalence of depression among caregivers in poor rural areas. For example, as part of a study of child development, Wei et al. (2015, 2018) found that around 40% of rural caregivers had depressive symptoms, and that these were a significant predictor of developmental delay in children. However, to our knowledge, no studies have attempted to identify the correlates of or risk factors for depression among caregivers in rural China. Understanding the extent to which depression is a problem among rural caregivers—and any potential consequences for the children that they are raising—is important for the development of targeted prevention services in rural areas, where mental health is less understood.

The purpose of our study is to gain a better understanding of depression among female primary caregivers of young children in rural China. To do so, we use a mixed methods approach that allows us to supplement quantitative data with qualitative reports. We use the quantitative data to present a large-scale snapshot of depression and its correlates in rural China, and then use interview-based qualitative data to conduct a deeper exploration of factors contributing to depressive symptoms. More specifically, we first report on the findings of a large-scale survey (*n* = 1,787) of depression among caregivers in the Qinling Mountain Region of rural China. Using this dataset, we report on the prevalence of depression, examine statistical correlations between caregiver depression and child development measures, and study individual-, household-, and community-level factors that may be linked with depression. Building on these findings, as well as findings from the existing literature on caregiver depression (introduced in more detail below), we go beyond the quantitative results by using a set of qualitative interviews (n = 51) to delve deeper into the factors contributing to depressive symptoms.

### Potential Risk Factors for Depression in Rural China

We conducted a detailed review of the literature—both in China and internationally—to identify factors that may be correlated with caregiver depression in rural areas such as rural China. There are many reasons why caregivers in rural areas might be especially prone to depression. One reason might be a lack of social support (Hou et al., 2015). Both instrumental (for example, practical and financial support) and emotional support are associated with better overall well-being and a lowered risk of depression (Israel et al., 2002; Stice et al., 2004; Moak and Agrawal, 2010). Research has shown that mothers who receive higher levels of social support have lower levels of maternal depressive symptoms (Bost et al., 2002; Cairney et al., 2003). Within the specific context of China, previous research has suggested that women who are “left behind” in rural areas, especially those geographically isolated from major cities, are more likely to suffer from psychological symptoms, including depression and anxiety (Qiu et al., 2016). Without social support from family or neighbors, daily life stressors can become isolating and at times overwhelming.

The general burden of caregiving may be another factor contributing to depressive symptoms. In rural China, primary caregiving of infants and toddlers typically falls to mothers or grandmothers, because of cultural norms, and because fathers often migrate to cities for work (Wang and Mesman, 2015). Moreover, many young mothers in rural China are left with the responsibility of caring for both their children and elderly in-laws. This excessive burden of caregiving has been linked with maternal mental health problems (Cheng et al., 2015).

For young mothers, evidence has also pointed to family conflict, such as disagreements with in-laws, as a persistent stressor within rural Chinese households (where the mother-in-law commonly resides together with the daughter-in-law). The dominance of the mother-in-law is rooted in Chinese Confucian society (Chan and Levy, 2004). In one study, nearly one third of rural women in China identified conflict with their mother-in-law as a major life event experienced prior to attempting suicide (Pearson et al., 2002). When asked to describe what they thought to be the dominant reason for the suicide attempt, both patients and their family members reported family conflict. While it should not be assumed that a high rate of suicide is equal to high rates of depression, Pearson's research nonetheless illustrates the acute life stress in rural households that is brought on by family conflict.

A fourth possible risk factor for depressive symptoms among rural caregivers may be a perceived lack of control or agency. A number of studies have supported the notion that depression is linked to the level of perceived control someone has over their life (Ross and Mirowsky, 1989, 1990). Rural women—especially young mothers living with their in-laws—may suffer from a perceived lack of control within their families. Given the traditional role of the mother-in-law as the dominant matriarch of the family (described above), young women in rural China, who are frequently isolated and have little income or education, may feel trapped in a social situation that is beyond their control (Kennedy, 2014).

## Methods

### Ethical Approval

Ethical approval for this study was granted by the Stanford University Institutional Review Board (IRB) (Protocol ID 35921). All subjects gave written informed consent in accordance with the Declaration of Helsinki. When participants were judged to be severely depressed and in need of help they were referred to the nearest hospital that provided mental health care services.

### Quantitative Sample Selection

Our quantitative study was conducted in seven nationally designated poverty counties located in China's Qinling Mountain Region. From each of these seven counties, all townships (the middle level of administration between county and village) were selected to participate in the study. There were two exceptions to this rule: we excluded the township in each county that housed the county seat, and we excluded any townships that did not have any villages with a population of 800 or more. In total, according to these criteria, 116 townships were included in the study.

The sample villages and households were selected as follows. From each township, we randomly selected one village to participate. Once the village was selected, a list of all registered births over the past 12 months was obtained from the local family planning official in each village. All children in our desired age range (6–24 months) were enrolled in the study. If a village had fewer than 10 children in our desired age range, we randomly selected an additional village in the same township for inclusion in the study and continued to randomly select additional villages until ten children per township had been found. Overall, our study uses data from 1,787 households in 118 sample villages (116 sample townships).

### Quantitative Procedure

Teams of trained enumerators collected socioeconomic information from all households participating in the study. The data used in this study were collected from sample households over two four-week periods in November 2015 and April 2016. All tests and survey instruments were administered one-on-one in each household. Each child's primary caregiver was identified and administered a detailed survey on parental and household characteristics, including each child's gender and birth order, maternal age and education, and whether the family was receiving Minimum Living Standard Guarantee Payments, a form of government welfare for the lowest income families nationwide. The exact age of each child was obtained from his or her birth certificate. The primary caregiver (the child's mother or grandmother in 1,670 out of 1,787 cases) was self-identified in each family as the individual who bears the most responsibility for the child's care. Indicators of geographical isolation were also collected, including the average number of households in the village and the average distance between each village and the nearest township.

#### Measuring Depression

All households were administered the Depression Anxiety and Stress Scale-21 (henceforth, DASS-21), a shortened version of the DASS-42, developed by Lovibond and Lovibond (1995). The DASS-21 is a self-report questionnaire, consisting of 21 items, that measures distress levels along three axes—depression, anxiety and stress—over the previous week. The DASS-21 cannot be interpreted as a categorical measure of clinical diagnosis (Lovibond and Lovibond, 1995), but is designed as a quantitative measure that assesses the severity of depression and anxiety symptoms.

Henry and Crawford (2005) found both adequate construct validity and high reliabilities of the DASS-21. Specifically, they found reliabilities were 0.88 for Depression, 0.82 for Anxiety, 0.90 for Stress, and 0.93 for the total scale. In addition, the authors established that the DASS-21 scales “contain variance that is specific to each scale” (p. 238), while also indexing “a substantial common factor (i.e., general psychological distress)” (p. 238). Chan et al. (2012) established the cross-cultural validity of the DASS-42 in China. Several years later, Wang et al. (2016) similarly established the validity for the DASS-21, also in China.

In our study, we focus solely on the depression scale, which considers hopelessness, self-deprecation, dysphoria, devaluation of life, inertia, and lack of interest and/or involvement. We follow the scoring method described by Lovibond and Lovibond (1995). The DASS-21 depression scale (with scores ranging from 0 to 63) comprises five levels of severity: normal (score: 0–9), mild (score: 10–13), moderate (score: 14–21), severe (score: 22–27), and extremely severe (score: 27+).

#### Measuring Child Development

All children were administered the Bayley Scales of Infant and Development, Third Edition (BSID-III), an internationally validated test of infant and toddler cognitive, language and motor development. This test is commonly used and is listed by the American Psychiatric Association as a standard way to diagnose certain developmental disorders (American Psychiatric Association, 2013). We assessed both cognitive and language development using the Bayley Scales of Infant & Toddler Development (BSID). More specifically, we administered the third—and most recent—edition of the test (BSID-III). The test directly assesses the child's performance on a series of tasks and produces standardized scores that take into account the child's gestational and chronological ages (Weiss et al., 2010). The cognitive scale assesses information processing (attention to novelty, attention to stimuli, and problem-solving), counting, and number skills whereas the language scale assesses receptive and expressive communication skills (Weiss et al., 2010). Studies examining the validity of the BSID-III found that the two scales exhibit high inter- and intra-rater reliability agreement, high internal consistency, and high test-retest stability even when tested in other cultural contexts (Zakaria et al., 2012; Yu et al., 2013; Madaschi et al., 2016; Azari et al., 2017). The inter-rater reliability, or Cronbach alpha, in our sample was over 0.95.

To assess the child's social-emotional development, we used the Ages and Stages Questionnaire: Social-Emotional (ASQ:SE), an internationally-recognized, scaled test of infant and toddler SE development (Squires et al., 2002). The ASQ:SE is a caregiver-completed questionnaire which evaluates the development of skills like self-regulation, compliance, communication, adaptive functioning, autonomy, affect, and interaction with other people. We used an officially-adapted Chinese version of the test (Bian et al., 2017). The ASQ:SE has an overall internal consistency of 0.81, test-retest reliability of 0.94, concurrent validity of 0.89, sensitivity of 0.82, and specificity of 0.92 (Squires et al., 2001). Psychometric properties were similarly reliable when tested in different populations (Kucuker et al., 2011; Heo and Squires, 2012; Chen, 2017).

The scoring of the ASQ:SE was done as follows. Caregivers were presented with a list of behavioral items in the survey, to which they respond with one of three rates at which their child displays a given behavior: “most of the time,” “sometimes,” or “never.” These behavioral items include, for example, “When upset, can your child calm down within 15 min?” and “Does your child laugh or smile when you play with her?.” Depending on the nature of the behavior and the age of the child, each response receives a numerical score. Because each age group has a slightly different scoring scheme, we standardized the ASQ:SE scores by age group (by subtracting the mean of a given age group and dividing by the standard deviation of that age group). Important to note here is that unlike the cognitive and language development scores, the score derived from the ASQ:SE is inversely related with development; in other words, a lower ASQ:SE score means better development.

### Statistical Analysis

In our study, we used the cognitive, language and social-emotional scales to measure children's development using the BSID-III. The scores for these scales are age-standardized with expected means (SD) of 105 (13) (Lowe et al., 2012; Bos, 2013; Jary et al., 2013; Serenius et al., 2013), 109 (15) (Bos, 2013; Jary et al., 2013; Serenius et al., 2013), and 100 (15) (Chao et al., 2011; Peng et al., 2013), respectively. Children are considered delayed in their cognitive, language or social-emotional development if they score at least one standard deviation lower than the mean on each of the respective scales (Anderson et al., 2010).

All statistical analyses were performed using STATA 14.0. *P*-values below 0.05 were considered statistically significant. STATA's multiple linear regression model was used to conduct the multivariate analysis. We included the following variables as potential confounders in the multivariate analysis: child's age, child's gender, whether the child was born prematurely, whether the child has siblings, whether the mother or grandmother is the primary caregiver, paternal and maternal age and educational level, whether the father lives at home, asset index (Principal Component Analysis score^1^), and whether the family received Minimum Living Standard Guarantee Payments. Community level factors included the average number of households in the village, and the average distance between the village and the township. In order to account for the nested nature of the data used in this study, we cluster all standard errors at the village level.

### Qualitative Procedure

A subset of primary caregivers from the quantitative sample described above were selected for more detailed follow-up interviews in March and April 2017. In total 55 participants were selected for our study: 27 mothers and 28 grandmothers. Four participants (three mothers, one grandmother) were excluded from analysis, since they were either unwilling or unable to participate in the interview. Our qualitative sample therefore includes 51 caregivers from 14 villages in four counties.

Individual participants were chosen for qualitative follow-up based on their scores on the DASS-21 during the quantitative study. We selected caregivers with scores placing them in either the top quartile (most depressed) or bottom quartile (least depressed) of the depression scale of the DASS-21. The two groups were labeled as the *non-depression group* and the *depression group*. The non-depression group (*n* = 27) consisted of participants who scored lowest (least depressed) within the normal category on the DASS-21 depression scale. The depression group (*n* = 24) consisted of participants who scored highest (most depressed) within the moderate (*n* = 17), severe (*n* = 4), or extremely severe categories (*n* = 3). We included both mother and grandmother caregivers in our sample, aiming for a 50–50 breakdown in both the non-depression group and the depression group.

#### Hypotheses

The purpose of the qualitative research was to understand “Why are caregivers depressed?” Interviews were conducted with the mothers and grandmothers of young children, following an interview protocol described in more detail below. Hypotheses were generated through an extensive literature review, presented above in the section titled “Potential risk factors for depression in rural China.” Our hypotheses were organized into four different topic groupings, each with a fixed set of open-ended questions. First, we hypothesized that lower levels of social support would be associated with depression. Questions aimed at understanding the level of social support focused on the presence of and contact with family or friends nearby, and the caregiver's ability to depend on others in terms of practical or emotional support. Second, we hypothesized that a larger caregiving burden would be associated with depression. Caregivers' perception of the burden of childrearing was assessed by discussing topics related to the time and effort involved in caring for the child and / or other adults in the family, as well as the level of stress that caregivers experienced. Third, we hypothesized that within-family conflict would be associated with depression. Local cultural factors that might influence this, such as mother-in-law daughter-in-law conflict (Zheng and Lin, 1994), were considered. Finally, we hypothesized that a perceived lack of agency would be associated with depression. Questions aiming to understand a caregiver's degree of control over their lives and their household included questions about their decision-making power in the household, and whether they felt their voice was heard within the family.

#### Data Collection for the Qualitative Interviews

The qualitative interview consisted of three parts. In the first part, updated background information was collected from all participants (Table 1). In the second part, the DASS-21 was re-administered to participants (12–18 months after the first administration during quantitative data collection) and participants were asked about their current mental state and the specific symptoms they reported experiencing on the DASS-21. If participants scored in a different category of the depression scale on the DASS-21 than they did during the previous administration of the DASS-21 (at the time of the quantitative survey), they were asked about their state of mind both at the time of the qualitative interview and during the last DASS-21 administration, as well as about the factors involved in this change.

**Table 1:** Descriptive statistics of caregiver depression, child developmental outcomes and family background characteristics, *n* = 1,787.

	**All Sample**	
**Variables**	**Mean**	**Std. Dev**.
**CAREGIVER DEPRESSION AND CHILD DEVELOPMENTAL OUTCOMES**		
Caregiver depression (1 = yes; 0 = no)	23.5	0.4
*Depression among mother caregivers (1 = yes; 0 = no)*	18.9	0.4
*Depression among non-mother caregivers (1 = yes; 0 = no)*	34.7	0.5
Cognitive delay (1 = yes; 0 = no)	37.6	0.5
Bayley mental development index (MDI)	95.9	12.6
Language delay (1 = yes; 0 = no)	52.0	0.5
Bayley language development index (LDI)	92.4	13.4
Social-emotional delay (1 = yes; 0 = no)	46.2	0.5
**CHILD CHARACTERISTICS**		
Age of child (months)	14.4	5.5
Female (1 = yes; 0 = no)	48.3	0.5
Premature birth (1 = yes; 0 = no)	4.9	0.2
Siblings (1 = yes; 0 = no)	51.9	0.5
**HOUSEHOLD CHARACTERISTICS**		
Mother is primary caregiver (1 = yes; 0 = no)	68.3	0.5
Grandmother is primary caregiver (1 = yes; 0 = no)	25.2	0.4
Maternal age (year)	27.9	4.9
Grandmother age (year)	54.3	6.9
Primary caregiver educational level (years)	8.1	3.4
Maternal educational level (1 = 12 years or above; 0 = else)	20.9	0.4
Paternal educational level (1 = 12 years or above; 0 = else)	25.7	0.4
Grandmother educational level (1 = 9 years or above; 0 = else)	3.7	0.2
Father lives at home (1 = yes; 0 = no)	44.2	0.5
Asset index (PCA score)	0.006	1.2
Household receives government welfare (1 = yes; 0 = no)	10.9	0.3
Number of households in the village	549.0	263.0
Distance between village and town (km)	5.5	6.4

*Source: Authors' survey*.

In the third part of the qualitative survey, semi-structured interviews were conducted. Caregivers were asked a series of open-ended questions about general aspects of their life in accordance with the study hypotheses (discussed above). A written interview protocol served as the primary guide. Interviewers ensured that all sections were covered, and probed for additional details when other topics related to the mental state of the participant came up. The interview concluded by asking participants a final set of questions: whether they had ever heard about mental health or depression, and, if yes, what their opinions or thoughts were on these matters. The full interview protocol is available from the authors upon request.

Interviews took place at the homes of the participants where caregivers were able to talk privately. Interviews lasted 1 to 2 h. All interviews were conducted by the same research team in Mandarin Chinese and the local dialect. All interviewers were trained in qualitative interviewing methods, and one interviewer was trained in clinical psychology.

#### Data Analysis

Interview data were de-identified, then analyzed and coded by the same research team that collected the data. The coding process was conducted in two stages. First an inductive coding method was used to identify and organize the data into general themes. Researchers looked for sentences or words in the interviews that could indicate a larger underlying topic or theme. These specific phrases or words were then gathered in order to see which phrasings could be grouped into a similar theme. Subsequently, the researchers counted in how many of the interviews these themes appeared. At this stage of coding, special attention was paid to both new themes that emerged from the interviews, as well as those that were derived from the initial interview framework. Second, the non-depression and depression groups were compared based on how often they mentioned specific themes in relation to their emotional state. The coding was done independently and subsequently cross-checked by two researchers in order to ensure validity and consistency of results.

## Results

### Quantitative Results

Basic socioeconomic and demographic characteristics of study participants are reported in Table 1. Mother primary caregivers are 28 years old on average (range: 16–57 years old), while grandmother caregivers are 54 years old on average (range: 33–88 years old). The average age of children in our sample is 14.4 months (range: 6–24 months). Of the primary caregivers, 68.3% (1220/1787) are mothers, 25.2% (450/1787) are grandmothers, and 6.5% (117/1787) are other family members (fathers, grandfathers, aunts or uncles). In this paper, we exclude “other family member” caregivers from our sample in order to focus on primary caregivers who are mothers and grandmothers, since these account for the vast majority (95%) of our sample. A total of 20.9% of mothers in our sample have completed high school or above, while only 3.7% of grandmothers in our sample have completed middle school or above. Just under half (44%) of fathers live at home. About 10.9% of sample families receive government welfare.

Village-level characteristics are also presented in Table 1. On average, there are 549 households per village in our sample (range: 150–2,103 households per village). The mean distance between sampled villages and their respective townships is 5.5 kilometers (range: 0.3–66 kilometers).

#### Prevalence of Depression

Our data show that 23.6% (421/1,787) of all primary caregivers are depressed, defined as scoring in the mild or higher category on the depression scale of the DASS-21 (Table 2). Of the depressed sample, 44.9% (189/421) of primary caregivers scored within the mild category, 42.3% (178/421) scored within the moderate category, 8.8% (37/421) scored within the severe category, and 4.0% (17/421) scored within the extremely severe category.

**Table 2:** Depression severity among depressed caregivers, *n* = 421.

**DASS severity**	**All depressed**	**Depressed**	**Depressed**
**ratings**	**caregivers (%)**	**mothers (%)**	**grandmothers (%)**
Mild	44.9	50.2	33.8
Moderate	42.3	38.5	47.4
Severe	8.8	9.1	9.0
Extremely Severe	4.0	2.2	5.8

*Source: Authors' survey*.

When we compare depression prevalence between grandmothers and mothers, the evidence suggests that depression may affect a larger proportion of grandmothers than mothers (*p* < b0.01). Overall, 18.9% (231/1220) of mothers and 34.7% (156/450) of grandmothers are depressed (mild or higher). Of the depressed mothers, 50.2% were mildly depressed, 38.5% were moderately depressed, and 11.3% were severely or extremely severely depressed. Of the depressed grandmothers, 33.8% were mildly depressed, 47.4% were moderately depressed, and 14.8% were severely or extremely severely depressed.

#### Child Development Outcomes: Cognitive, Language, and Social-Emotional Development

BSID-III measures are available for all 1,787 children (Table 1). In total, we find that 37.6% of sample children are significantly delayed in their cognitive development. Over half of sample children present delays in language development (52.0%). Nearly half (46.2%) of sample children are significantly delayed in their social-emotional development.

#### Relationship Between Caregiver Depression and Child Developmental Delay

Table 3 shows the association between caregiver depression and child developmental delay, after adjusting for potential confounders. We find a significant link between children's social-emotional delay and caregiver depression. Specifically, caregiver depression is associated with a 0.53 SD worsening in children's social-emotional development (*p* < 0.01). Caregiver depression is also associated with a 1.56 point (0.12 SD) decrease in children's language development (*p* < 0.05). There is no significant link between caregiver depression and children's cognitive development.

**Table 3:** Association between caregiver depression and child developmental outcomes using OLS, n = 1,787.

**Independent variables**	**Caregiver depression (1 = yes; 0 = no)**
	**OLS**
Cognitive development (test points)	0.375
	(0.670)
*N*	1,787
Language development (test points)	−1.562^**^
	(0.761)
*N*	1,787
Social-emotional development (SD)	0.529^***^
	(0.065)
*N*	1,787
Cohort dummies	Y
Controls	Y

*Source: Authors' survey*.

*Control variables are child's age, child's gender, whether the child was premature, whether the child has a sibling, migration, caregiver type, age of primary caregiver, educational level of primary caregiver, educational level of father, asset index, whether the household receives government welfare, the number of households in the village, and distance between the village and the township*.

** *significant at 5%*;

*** *significant at 1%. Robust standard errors in parentheses clustered at village level*.

#### Correlates of Caregiver Depression

In Table 4, we present the individual and household correlates of caregiver depression. We find that grandmother caregivers (*p* < 0.01) and caregivers living in poorer households (*p* < 0.05, as measured by an asset index) are more likely to be depressed. There is no significant link between caregiver depression and any of our other included variables: age of the child, gender of the child, premature birth, whether or not the child has siblings, primary caregiver's age, and education level, father's education level, whether the father lives at home, or whether the household receives government welfare. Also, no link was found between caregiver depression and either the average number of households in sampled villages, or the distance between the sampled village and the nearest township, both indicators of geographic isolation.

**Table 4:** Correlates of caregiver depression, n = 1,787.

**Independent variables**	**Caregiver depression (1 = yes; 0 = no)**
	**OLS**
**PANEL A: CHILD CHARACTERISTICS**	
Age of child (month)	0.002
	(0.002)
Female (1 = yes; 0 = no)	−0.026
	(0.019)
Premature (1 = yes; 0 = no)	0.056
	(0.047)
Has siblings (1 = yes; 0 = no)	0.008
	(0.024)
**PANEL B: HOUSEHOLD CHARACTERISTICS**	
Primary caregiver type (mother = 1; grandmother = 0)	−0.098^***^
	(0.033)
Age of primary caregiver (years)	0.001
	(0.001)
Primary caregiver educational level (years)	−0.007
	(0.004)
Father educational level (years)	−0.003
	(0.004)
Father lives at home (1 = yes; 0 = no)	−0.026
	(0.020)
Asset Index (PCA score)	−0.028^***^
	(0.009)
Household receives government welfare	−0.027
	(0.031)
Number of households in the village	−0.0001
	(0.0001)
Distance between village and township (km)	0.003
	(0.002)
Cohort	−0.003
	(0.014)
Constant	0.353^***^
	(0.092)
Observations	1,787

*Source: Authors' survey*.

^**^significant at 5%;

*** *significant at 1%. Robust standard errors in parentheses clustered at village level*.

As part of our effort to further examine potential contributing factors, we now use a set of qualitative interviews with mother and grandmother caregivers to explore caregiver depression on a deeper level.

### Qualitative Results

As part of our sampling strategy for the qualitative study, we visited 24 caregivers who had been depressed at the time of the quantitative survey, and 27 caregivers who had not been depressed at the time of the quantitative survey. In re-administering the DASS-21, we found that some previously depressed caregivers were no longer depressed (*n* = 12), and some previously non-depressed caregivers were now depressed (*n* = 4). In total then, at the time of our qualitative interviews, 16 caregivers were depressed and 35 participants were not depressed. Of the 16 depressed participants, 13 were grandmothers (81%), and 3 were mothers (19%). Of the 35 not depressed, 14 were grandmothers (40%), and 21 were mothers (60%). Qualitative analysis was based on depression scores of participants at the time of our follow-up interviews.

General descriptive data of our qualitative sample is presented in Table 5. On average, mothers are 28 years old (range: 20–36), and grandmothers are 54 years old (range: 47–63). The majority of caregivers (96.1%) are married (with the exception of two who are widowed). Caregivers have an average of 1.7 children to look after, with more than half of caregivers (60.8%) having more than one child to raise. Over half (52.9%) of caregiver-participants in our qualitative sample have an educational attainment of middle school or above. Notably, 15.7% of caregivers in our sample have no education. The vast majority of caregivers (92.2%) have at least one family member who is working outside the village. In a little more than half of our families (52.9%), the husband is reported to be living at home. A total of 40% of caregivers have worked before, compared to the majority (60.8%) who have not. Finally, 52.9% report having access to social media (through WeChat). All participants are of Han ethnicity.

**Table 5:** Socio-demographic information of qualitative sample, n = 51.

		**Not depressed**	**Depressed**
**Variable**	***n*(%)**	***n*(%)**	***n*(%)**
**CAREGIVER**			
Mother	24 (47, 06)	21 (60, 00)	3 (18, 75)
Grandmother	27 (52, 94)	14 (40, 00)	13 (81, 25)
**MARITAL STATUS**			
Married	49 (96, 08)	34 (97, 14)	15 (93, 75)
Widowed	2 (3, 92)	1 (2, 86)	1 (6, 25)
**NUMBER OF CHILDREN TO TAKE CARE OF**			
One	20 (39, 22)	11 (31, 43)	9 (56, 25)
More than one	31 (60, 78)	24 (68, 57)	7 (43, 75)
**EDUCATION**			
No education	8 (15, 69)	3 (8, 57)	5 (31, 25)
Illiterate	5 (9, 80)	0 (0)	5 (31, 25)
Primary school	16 (31, 37)	11 (31, 43)	5 (31, 25)
Middle school	23 (45, 10)	18 (51, 43)	5 (31, 25)
High school	4 (7, 84)	3 (8,57)	1 (6,25)
**FAMILY MEMBER WORKING OUTSIDE THE VILLAGE**			
Yes	47 (92, 16)	33 (94, 29)	14 (87, 50)
No	4 (7, 84)	2 (5, 71)	2 (12, 50)
**FATHER LIVES AT HOME**			
Yes	27 (52, 94)	17 (48, 57)	10 (62, 50)
No	22 (43, 14)	17 (48, 57)	5 (31, 25)
**WORKED BEFORE**			
Yes	20 (39, 22)	17 (48, 57)	3 (18, 75)
No	31 (60, 78)	18 (51, 43)	13 (81, 25)
**ACCESS TO SOCIAL MEDIA (WECHAT)**			
Mother	22 (43, 14)	20 (57, 14)	2 (12, 5)
Grandmother	5 (9, 80)	1 (2, 86)	4 (25, 00)
**ETHNICITY**			
Han	51 (100)	35 (100)	16 (100)
Totals (*N = 51*)	51	35	16

Following data collection, we conducted a general inductive analysis of interviews, identifying common “contributing factors” reported. These factors are defined in detail in Table 6. We identify six contributing factors: our four originally hypothesized factors, plus two unexpected findings. All are presented in detail below.

**Table 6:** Contributing factors of caregiver depression.

**Factors**	**Definition**	**Depressed (%)**	**Not depressed (%)**
**LACK OF SOCIAL SUPPORT**			
Social stigma associated with talking about negative feelings or difficulties within family	answered “no one,” when asked if and who caregivers talked to about worries, feeling annoyed or upset	5/16 (31%)	11/35 (31%)
Heard about mental health	answered positively to the question “Have you ever heard about mental health or depression?”	4/16 (25%)	17/35 (49%)
**BURDEN OF CHILDCARE**			
Child rearing as a burden	answered positively to the question “Is raising a child a burden?”	8/16 (50%)	7/35 (20%)
Badly behaved children	children are said to be “badly behaved,” “naughty,” “don't listen very well,” “not well behaved”	5/16 (31%)	10/35 (29%)
Hitting their child	hitting the child is mentioned, often in combination with “being annoyed,” “in a bad mood.”	5/16 (31%)	7/35 (20%)
Child as protection	child(ren) are actively mentioned as protecting the caregiver from feeling lonely or helping the caregiver to feel better	7/16 (44%)	9/35 (26%)
**WITHIN-FAMILY CONFLICT**			
Presence of conflict within the family	mentions such as “fight,” “conflict,” “disagreements,” “arguments”	8/16 (50%)	12/35 (34%)
Arguments with spouse	specific arguments with the spouse were named	6/16 (38%)	10/35 (29%)
Conflict with daughter-in-law	only grandmothers; when complaining about the daughter-in-law for not doing what she is expected to do (e.g. not sending money, not caring well for the child, fighting or leaving the husband)	5/13 (39%)	2/14 (14%)
Conflict with mother-in-law	only mothers; when complaining about the mother-in-law for not doing what she is expected to do (e.g. raising the children)	0/3 (0%)	3/21 (14%)
**LACK OF CONTROL AND AGENCY WITHIN HOUSEHOLD**			
Feeling worthless	answered positively on the item “I felt that I wasn't worth much as a person” of the DASS-21	13/16 (81%)	9/35 (26%)
Illiteracy	“cannot read,” “cannot write,” “cannot recognize characters,” “illiterate”	5/16 (31%)	0/35 (0%)
Old age being a reason for lack of worth	only grandmothers; when old age is given as a reason for feeling worthless	3/13 (23%)	0/14 (0%)
**POVERTY**			
Lack of money	mentioning loans that are difficult to pay off, not being able to buy important things, money being tight, needing more money	14/16 (88%)	12/35 (34%)
Illness	mention of “sick,” “ill,” “illness,” “pain,” “hurt,” “in bad health”	13/16 (81%)	14/35 (40%)
Material wealth as measure of self-worth	when comparing with other families in terms of material wealth is mentioned as a reason for one's own worth	11/16 (69%)	12/35 (34%)
Totals (*N* = 51)		16	35

#### Hypothesized Factor 1: Lack of Social Support

Our first hypothesis was that lower levels of social support would contribute to depression. Indeed, we observe a strong social stigma around discussing personal problems with others in the village. While many women do not talk about their troubles at all, even those women who do are typically not receiving meaningful support that allows them to deal with their problems constructively.

The idea of talking to someone when feeling troubled is not common practice in rural China. In general, 16 out of all 51 participants (31%) do not talk to anybody when they feel angry or upset. Different reasons are given for this, ranging from “you don't talk about personal things outside the family,” to “I don't want others to worry about me” or even “I don't have anyone to talk to.” While we found no significant difference between depressed and non-depressed caregivers in this regard, this social trend may be more important for depressed caregivers, as it may exacerbate existing negative feelings.

I don't have any [friends] to talk to…

– A16 Depressed grandmother

I don't usually [talk about it], I keep it inside. In the village, we have a saying: ‘don't show your worries from inside the family to the outside world.' In general, you just endure bad times by yourself… If you tell people about [your troubles], other people will laugh at you. If you tell them about your troubles… they will spread the news around.

– B23 Non-depressed grandmother

I'll just stay by myself for a while and then I will calm down. If I talk to my parents [about my mood], they'll just worry about it…so I don't talk to them.

– B27 Depressed mother

When something is agitating me, there is no point in telling anybody about it, because then we will be agitated together.

– A24 Non-depressed mother

While there is some degree of practical support (e.g., looking after a neighbor's child) we do not find evidence of a high degree of emotional support in the villages. Although many (35/51 or 69%) mention having someone to talk to, comfort from friends or family seemed to be largely provided in the form of empty phrases and general sayings. For example, when asked how friends and family comforted them, many caregivers respond with phrases such as “don't think about it,” “think about happier things,” or “everyone has this.” This may suggest that a common way people deal with their problems is by ignoring them, rather than dealing with them or talking about things in a meaningful way. Most caregivers mention that they mainly talk about safe and neutral subjects—like their children—with friends and neighbors in the village.

[Interviewer]: Do you talk to your husband or children when your mood is bad?

[Grandmother B17]: Yes, I talk to them about it.

[Interviewer]: How do they react?

[Grandmother B17]: What can they say, they just listen… they just say, “you think too much.” And then I think to myself, I shouldn't talk to anybody about this…

– B17 Depressed grandmother

This reserve in talking to others may in part be reinforced by the assumption that other people would not understand when trying to talk to them. Many caregivers express a sense of futility when attempting to talk to somebody about their mood.

I used to feel bad and try to talk to my [husband] about it. But there's no point in saying anything, he won't understand anyway.

– B6 Non-depressed mother

When you talk to somebody about what's bothering you, it's not like they can solve your problems for you.

– B2 Non-depressed grandmother

[My husband] just didn't understand why I felt so bad. There were plenty of mothers who were fine and didn't feel that bad.

– B21 Non-depressed mother (previously had postpartum depression)

Our interview results show that another reason why people are reluctant to share problems or feelings of unhappiness with neighbors or friends is because it is stigmatizing to have something “wrong” with you. Caregivers often express a fear of people gossiping or “losing face” at the prospect of others knowing of an illness in the family.

[I feel I don't have much worth]. Other families look down on you if there is something wrong with your family. For example, when my daughter (who has cognitive problems) says something, and she doesn't say it in a way that is comprehensible, the people in the village will look down on her. When I see people doing this to her, as a mother, how do you think I feel inside?

– B23 Non-depressed grandmother

[My friends] will talk about [my daughter's illness] with other people and they [will] laugh at me. Aren't I losing face?…We've spent 450,000 RMB trying to cure her…Everyone laughs at her, and they'll say things like “Why is your child so ugly?” Who wants to hear that? I'm worried about it every day and I can't calm down… in front of other people, I feel so ashamed. We've spent so much money and her skin hasn't healed. I feel very inferior.

– B27 Depressed mother (daughter has a rare genetic skin disorder)

They say mean things [about my illness] behind my back. They say that I did something during my last life, and that's why I got this illness, because I did something bad.

– B24 Non-depressed mother with previous illness

My husband had meningitis and now he is disabled. I feel different from other households; I worry that other people will laugh at my family. Because my husband is disabled, he can't go out to work and earn money.

– A9 Non-depressed grandmother

In these villages, you only go to the hospital for something physical, otherwise they think that you're fragile…[Mental health issues] are things you need to deal with by yourself.

– B21 Non-depressed mother (previously had postpartum depression)

The concept of mental health is often stigmatized and not well-understood. Only 21 out of 51 interviewed caregivers (41%) have ever heard of depression or mental health before. Among those who have heard of it, depression (including postpartum depression) is described as “crazy” behavior, such as “standing naked in freezing water,” “doing stupid things,” or violence resulting in suicide or death (e.g., women killing their own children). When asked what they understand by depression, many participants provide extreme examples of their impressions.

Some people have said that you can get postpartum depression after having a baby… some women have even killed their children. So [depression] sounds very scary.

–B6 Non-depressed mother

A few years ago, a young man killed somebody. And he had depression…A stranger came in his home and the man [with depression] killed him.

–B3 Non depressed grandmother

When caregivers indicate that they know what depression is, their answers often suggest that their understanding of depression is limited.

People who are physically healthy are also mentally healthy.

– B18 Non-depressed grandmother, when asked to define mental health

[Interviewer]: Have you ever heard of depression or mental health?

[Mother B19]: Yes…I've only heard that if you're not feeling well inside, you should go see a counselor.

[Interviewer]: What about depression? Have you heard about depression?

[Mother B19]: It's like autism, right?

–B19 Non-depressed mother

[Interviewer]: Have you ever heard of depression or mental health?

[Mother A17]: Yes, I've heard about it on television.

[Interviewer]: What is your understanding of it?

[Mother A17]: Someone who locks himself inside his home… and when other people want to talk to him, he doesn't want to talk. Sometimes the person [with depression] will talk to himself.

– A17 Non-depressed mother

Notably, caregivers who have heard of depression described that they had learned about these concepts through television programs, or through social media such as WeChat. For example, one young mother recognized the symptoms of postpartum depression in herself after filling out a test on social media (posted by a friend on WeChat) and consequently opted to leave home for two months in order to “get out of the house” and to “change [her] environment.” This suggests that forms of social media could potentially be useful in spreading information and awareness about mental health in rural areas.

[Interviewer]: Have you ever heard of depression or mental health?

[Mother B21]: Yes…because I've had it myself and I know what it is.

[Interviewer]: How do you know that you've had it?

[Mother B21]: I used Baidu [an online search engine] to find out what [depression] was after I scored positive on a test. After I knew, I felt that I needed to change my temper.

[Interviewer]: How did you know to test yourself?

[Mother B21]: I was scrolling through WeChat 1 day and my friend posted a short quiz about depression. I tested myself. After this, I paid more attention to [my depression] and I tried to make myself feel better.

– B21 Non-depressed mother (previously had postpartum depression)

#### Hypothesized Factor 2: Burden of Caregiving

Depressed caregivers are more likely to report childrearing as a significant burden. As many as 8 out of 16 depressed caregivers (50%) answered positively when they were asked if they thought raising children was a burden, vs. 7 out of 35 non-depressed caregivers (20%).

The burden of raising children mostly comes from the exacerbation of existing financial or physical issues. For example, expenses associated with buying infant formula, sending children to school and doctor visits, are often brought up.

When I think about the things that don't allow me to relax, then I think about the children that haven't grown up and I'm getting older and the child needs to drink infant formula and I don't have the money [to buy this for the child].

– A18 Depressed grandmother

The burden of caregiving appears to fall heaviest on older caregivers. All depressed caregivers who mentioned caregiving as a burden are grandmothers, except for one mother (who took care of three children on her own). Grandmothers experience more of a physical burden or physical discomfort from taking care of their young grandchildren, often getting tired from chasing after them or having trouble holding the child due to existing issues of chronic pain.

I'm almost 70 years old, taking care of children is not easy … after my youngest grandchild's mother left, I've begun to feel that with every day that I need to take care of the child, I get more agitated. I'm old, I can't do anything… I need people to take care of me, but I still need to take care of these children.

– A18 Depressed grandmother

It's difficult for me to chase after the children because my legs will hurt.

– B5 Depressed grandmother

This little one [the youngest grandchild] is more tiring [to take care of]. I'm old now and it's very difficult to hold him. Sometimes I can't pick him up and I need to use the little toy car to push him around.

– A18 Depressed grandmother

Sometimes I feel overwhelmed with taking care of my grandson. He is very clingy! He always wants to be held and then I feel very annoyed, and angry… [I would feel like] I don't want to take care of the child… When I feel like this I just lie on the bed, and then I don't want to eat… Every week this happens at least once and then I need to sleep for a few hours… Sometimes it feels unbearable.

– A8 Depressed grandmother

Depressed caregivers are more likely to hit their children, and mentioned being more prone to do so when angry or frustrated. Of 16 depressed caregivers, 5 report hitting children when their mood is bad (31%), compared with only 7 out of 35 non-depressed caregivers (20%). However, it does not seem to be the case that these children of depressed caregivers are more misbehaved, as having unruly children is mentioned by depressed (5/16 or 31%) and non-depressed (10/35 or 29%) participants equally in interviews. This suggests that depressed caregivers are more susceptible to feeling frustrated due to stress from caregiving.

I will hit her [my granddaughter] when I'm in a bad mood, because she's always running away.

– B5 Depressed grandmother

The burden of caregiving applies not just to child care, but to elder care as well. Conflict often arises when a member of the family falls seriously ill, and others need to take on an extra burden of care, including caring for the sick family member and taking over their housework. Arguments over these extra responsibilities frequently lead to relational and emotional stress within the family.

For example, spousal conflict regularly emerges when partners argue over how to divide the care of their children or elderly parents. Depressed caregivers report frustration with their spouses at a higher rate than non-depressed caregivers (6/16 or 38%, vs. 10/35 or 29%).

Taking care of my mother-in-law is another thing [we fight about]. I want to take care of my mother-in-law myself but my husband thinks it's too tiring for me and that we should hire somebody to take care of her…

– B10 Depressed mother

I have to worry about a lot of things, my husband doesn't need to care about anything… If only someone would help me. My son is outside working; my son's wife also needs to be taken care of. She has epilepsy. My husband doesn't care. No one helps me.

– B13 Depressed grandmother

Sometimes I fight with my husband, and then afterwards I feel sad. We fight because of the child… if she gets sick or hurt her father will blame me and then we'll fight.

– B28 Depressed mother

When the child got sick and I called my husband [who works in Beijing] …he asked, “Why are you calling me? Go call the doctor.”

– B9 Depressed grandmother

Despite the challenges of childrearing, many caregivers also mention children as a protective factor against negative feelings such as sadness or loneliness. Interestingly, more depressed caregivers than non-depressed caregivers mention that they view their children as a protective factor (44 vs. 26%).

Ever since I've been taking care of the child, it's been easier… [After my son's death], my granddaughter helps me have fun. As long as I have her, I feel better. I can relax, I worry less…

– B4 Depressed grandmother

Have you seen my hair? It's completely white. All because of worrying! But taking care of my grandchild makes me happy. When you're old, all you hope for is that you can look forward to having a grandchild.

– B13 Depressed grandmother

My son and daughter-in-law wanted to take my grandson with them to the city [to raise their child themselves] … but I didn't let them, because if I didn't have him I would be alone and too lonely.

– B20 Depressed grandmother

#### Hypothesized Factor 3: Within-Family Conflict

Depressed caregivers (8/16 or 50%) mention the presence of conflict in their households more often than their non-depressed counterparts (12/35 or 34%). Commonly reported topics of family conflict allude to others in the family (parents, in-laws, husband, daughter-in-law) refusing to help with farm work, housework or childcare.

I am very easily angered, I want my relatives to help me with the farm work, harvest the potatoes, plant the corn… but my family doesn't come…. And no one helps me with taking care of the child.

– A8 Depressed grandmother

Sometimes, yes, I feel [down-hearted]. Because of daily life at home…there isn't anyone to take care of the children… the children are young, the elders are very old.

– A18 Depressed grandmother

As described previously, we did not find many mothers who reported feeling constrained or agitated due to conflicts with their mother-in-law (despite previous research that suggests this). Even when one mother described a particularly strained relationship between her neighbor and her mother-in-law, the conflict dissipated quickly once the mother migrated to the city with her child. Another mother similarly relayed her eventual plan to migrate to a city, mostly to seek better schooling for her child.

[Mother A23]: There was a really serious conflict between my neighbor and her mother-in-law. The mother would hit her child and the mother-in-law would get very angry. They would start fighting about it. When I moved here, I could hear them fighting. Sometimes I would see them hitting each other, every 3 to 4 days.

[Interviewer]: How was it resolved?

[Mother A23]: The mother moved to the city with the child.

– A23 Non-depressed mother

I plan to wait until the child is 1 year old… then bring him with me to the city to attend school.

–A25 Non-depressed mother (who reports a bad relationship with her mother-in-law)

Depressed grandmothers however often express annoyance with their daughter-in-law for not doing what she is expected to do. For example, more depressed grandmothers (5/13 or 39%) than non-depressed grandmothers (2/14 or 14%) express discontent with their daughter-in-law. Reasons given for this include not sending home money for the child while working far away, not getting pregnant, not knowing how to properly take care of the child, or fighting too much with the husband (their son). Interestingly, this is not found in the other direction: rarely does the daughter-in-law complain about the mother-in-law not fulfilling a certain role.

My daughter-in-law told me not to buy any cheap things for the child, but when [the daughter-in-law] comes home, she doesn't even buy him anything, not even candy. And if I want money [for the child], my daughter-in-law won't give it to me.

– B20 Depressed grandmother

[I worry about] my eldest son's marriage, because my daughter-in-law hasn't gotten pregnant and it's been 5 years. I feel very stressed, because I can only think about these matters. I can't let it go.

– A3 Depressed grandmother, when asked about her biggest worry

#### Hypothesized Factor 4: Lack of Control and Agency Within Household

In spite of prior research that suggests that many younger caregivers in rural Chinese households experience depression due to loss of a “control” or decision-making power to the mother-in-law in the household (Chan et al., 2009), we did not find evidence of this as a source of distress. Young mothers—both depressed and otherwise—generally expressed contentment with the current state of decision-making in the household, and did not explicitly express wanting more control, or feeling limited by their role.

[Mother B24]: My mother-in-law is dominating. She likes to be the decision- maker.

[Interviewer]: How do you feel about this arrangement?

[Mother B24]: I think it's quite good, because I'm someone who can't really make decisions. So I think it's quite good [this way].

– B24 Non-depressed mother

Sometimes I think that it's best for my mother-in-law to control the money, because I think that she has more experience than me. So, it's better for her to decide these things.

– B28 Depressed mother

By contrast, we find that many grandmothers often express distress and feelings of uselessness due to an inability to carry out their expected role in the household or be useful to their family. Regular feelings of low self-esteem are reported by 13 out of 16 depressed caregivers (81%), who typically use phrases such as “not having any worth,” “feeling useless,” “hating oneself,” or similar expressions. In comparison, only 9 out of 35 non-depressed caregivers (26%) mention feelings of low self-esteem (see Table 6). This discrepancy between depressed caregivers and non-depressed caregivers is to be expected as low self-esteem is a symptom of depression. When asked to elaborate on these feelings, many caregivers related their low self-esteem to being unable to help relieve the family's financial burden or provide adequate childrearing support. The ability to live up to these social expectations, either through finding work or raising children, appears to be a crucial factor to self-worth and feeling purposeful for caregivers in rural China.

Elderly caregivers frequently cite three specific reasons for feelings of worthlessness: illiteracy, old age, and illness. These three factors, combined with a general inability to change their circumstances, contribute to their perceived lack of agency in the home. First, illiteracy is frequently mentioned as a reason why caregivers feel a lack of purpose in their life. It is notable that all five illiterate caregivers in our sample are grandmothers who are depressed. Caregivers describe their illiteracy as something that prevents them from finding work and “easing the burden of the family.” However, we find that of all the grandmothers we interviewed, only two (2/27) had ever held a job, indicating that not having a job is typical for all grandmothers, not just illiterate ones. This suggests that illiterate grandmothers may view “not being able to get a job” as a proxy for not being able to “measure up” in the modern world. Having never worked in areas other than farming (and with little potential to find work as an older person), grandmothers may also perceive their illiteracy as a marker of being “less” than others, and unable to fit in—particularly in an environment where many people pursue work in the cities.

I have never had any worth, ever since my mother gave birth to me. … I didn't go to school, I can't read, I can't recognize characters, so what worth do I have?

– B17 Depressed grandmother, illiterate

[I started feeling worse when] people around me started getting jobs, and I couldn't. And then I started to panic, because the people around me got jobs.

– B9 Depressed grandmother, illiterate

I constantly hate myself, because I feel useless, and I feel like I'm not able to do anything. I always need to ask someone to help me… No one has ever asked me for my help with something. Even if other people might need me, I cannot help them. I cannot… I look down on myself for it. I would like to work, but I am scared that someone will tell me that I can't [work]. I would like to work as a nanny, but last time I tried, I told them that I can't read or write so I didn't get the job. I've seen so many advertisements looking for nannies.

– B9 Depressed grandmother, illiterate

Old age is another factor that is often brought up as a reason for feelings of worthlessness. Grandmothers in our sample often attribute feeling useless to their old age, expressing frustration at being physically unable to work and help their family. For many grandmothers, being elderly means that there is less of a possibility to change or improve their situation.

I'm old and I'm not really useful.

– B13 Depressed grandmother

When you're young you can go to a city and work. But when you're old, what are you supposed to do?

– B4 Depressed grandmother

What kind of worth do you have when you're old? Sometimes I really feel that I have no use. When you're old, what kind of worth do you have? You can't earn any money… you have nothing to pass on to the next generation. I often feel in despair.

– B13 Depressed grandmother

Third, depressed caregivers who are seriously ill also struggled with feelings of worthlessness stemming from poor health. For these women, good health appears to be intrinsically related to their self-esteem. Being ill means that a caregiver cannot fulfill her role (e.g., taking care of children and doing housework) and contribute to her family in ways that she is expected to. In turn, not being able to fulfill this expected “role” can lead to caregivers feeling like a burden to the family, contributing to feelings of self-doubt and worthlessness.

[Self-worth] is related to my physical health. If you don't feel well, there's nothing you can do.

– B16 Depressed grandmother

Older caregivers also report high degrees of emotional stress from worrying about what will happen to their family members if they become too ill to care for them.

I'm scared of getting ill, my head hurts and I often get dizzy. I worry about what will happen to the child if I am ill. Sometimes I'm so dizzy that I can't get up, and then I think “what do I do now?”

– B20 Depressed grandmother

### Unexpected Findings

In the course of our qualitative interviews, two additional contributing factors emerged, beyond our original hypotheses. These are described in detail below.

#### Unexpected Factor 1: Poverty

Caregivers who are depressed are much more likely to mention poverty as a source of distress. To illustrate, 14 out of 16 (88%) depressed participants report experiencing trouble with paying back loans, money being too scarce or a major source of worry, and not being able to buy necessary things. In contrast, only 12 out 35 (34%) of non-depressed participants mention monetary concerns (see Table 6).

Sometimes when we don't have any money to use, when there is not enough money, I worry. Right now, we need to buy oil and salt…and when you don't have the money to buy these kinds of things, of course you panic.

– B17 Depressed grandmother

Our situation at home is difficult. Nobody is earning money and we are in major debt. My husband has a lot of physical problems (such as arthritis in the neck), and because he always has issues with his health, he can't do anything. He can't work and there's no money…Of course I feel I am close to panicking. My husband can't work, my family doesn't have any money. It's really difficult.

– A16 Depressed grandmother

My mood isn't good. I feel agitated. I just feel like I don't want to do anything… It's the burden of taking care of three children. Only their father is working… to support three children. Money is hard to find and work is hard to come by. And there is an 80-year-old grandmother, too.

– B10 Depressed mother

I am very agitated all day. I spend all day worrying because I don't have any money. And where there is no money in the house, and the child gets sick, it becomes very hard to calm down.

– B9 Depressed grandmother

Are families of depressed caregivers actually worse off, or do they only perceive themselves to be? Almost all caregivers (47 out of 51 or 92%) in our sample have at least one member of the family who is working in a city (see Table 5), suggesting an external source of income other than farming. Why is it then that half of our sample (26 out of 51) express worry or distress from a lack of money?

We do find that depressed caregivers are more likely to have experienced recent income shocks, such as the serious illness of a family member. This suggests that their poverty is real rather than perceived, as such income shocks easily exacerbate their poverty. Depressed caregivers report illness in the family (e.g., diabetes, arthritis, epilepsy, meningitis) twice as often (13/16 or 81%) as non-depressed caregivers (14/35 or 40%).

Expensive healthcare and medicine is found to be a common instigator of significant financial stress. Access to quality healthcare is often severely limited in rural areas. This puts a heavier burden on poor families, both in terms of time and money. They are forced to travel long distances with limited public transportation options in order to receive treatment in hospitals in large cities. Beyond the cost of this extensive travel, they also have to foot accommodation costs once they reach the cities, since making the return trip in a single day is typically impractical, if not outright impossible.

After my daughter-in-law had her baby, she had an epileptic fit. We came back home with the mother and the child, she stayed for 10 days and then we had to call an ambulance to take her to the hospital again. It was like she was dead. Our daughter-in-law's expenses are very large. Every year she needs to go to Beijing and see a doctor. When I was taking care of my grandson, she went three times. She needs to go to Beijing [ca. 1,000 km], and we need to pay for transportation, the doctor, living there for a week, eating… and none of it is covered by our medical insurance.

– B13 Depressed grandmother

My husband is also sick. He has kidney stones, but we don't have any money to go see a doctor. Doesn't that make you nervous? It hurts him a lot. Sometimes I'm just so agitated and anxious. Just in the last few years, we've had so many things… so many things… mostly just family members being sick … lots of illnesses here and there.

– B13 Depressed grandmother

#### Unexpected Factor 2: Material Wealth as Measure of Self-Worth

Related to poverty, we find that the perception that material wealth is an important measure of self-worth may also contribute to depression. Material wealth is often expressed as a measure of one's success, which is taken to be analogous with one's happiness. When asked about why other women in the village are happy, caregivers answered:

The women in that village over there, they're pretty happy because the government took their land and gave them a lot of money.

– A19 Non-depressed mother

To be honest it's mostly about money. When there's no money, they're not happy.

– A23 Non-depressed mother

[Other women in the village are unhappy] because they are like my family—with no money. There are no other reasons why they are unhappy.

– A16 Depressed grandmother

While recognition of the importance of material wealth appears to be universal—reported by depressed and non-depressed caregivers alike—depressed caregivers appear to be more affected by it. The majority of depressed caregivers (14/16 or 88%) mention not being able to pay back loans, buy material goods, wanting or needing more money, compared with only 12/35 (34%) of non-depressed caregivers (Table 6). Depressed caregivers often associate a lack of material assets with feeling inferior, and frequently compare their own family with others. “How much money one has” is defined in relation to what others in the village have. New houses, new furniture, and how well other children are doing (in terms of employment prospects and income), are some of the main life indicators against which families compare one another. More often than not, perceiving oneself as being poor or having less than others is linked to feelings of worthlessness, and sometimes even to a meaningless life.

When I compare myself with someone else, I feel like my life doesn't have that much meaning… My relatives have been renovating their house and buying cars…[they] don't have any loans to pay back, and I still haven't even returned the money that I owe.

– B10 Depressed mother

I just feel that I'm not as good as other people. Other people just always seem to be able to do things better… and I am not [able to]. I always compare myself with others. For example, other people's homes are beautiful and they have social power and lots of money. In terms of these social connections, we cannot keep up.

– B17 Depressed grandmother

Other people are able to be rich, and I have no money. When I go to their homes, I feel like life is meaningless because I'm poor. I just think it's meaningless.

– B20 Depressed grandmother

When there is no money, there is no worth. People who have money [are people] who have worth.

– A16 Depressed grandmother

## Discussion

In this paper we bring together both quantitative and qualitative evidence to provide insight into the prevalence and correlates of depression among female caregivers in China's Qinling Mountain Region.

Our findings reveal a high prevalence of depression among women in China's Qinling Mountain Region. We show that approximately one quarter (23.5%) of all primary caregivers in our sample are depressed. This is higher than the global average, which ranges from 13 to 21% (O'Hara and Swain, 1996; Coll et al., 2016; de Castro et al., 2017). While no data currently exist on depression among caregivers of small children in urban China, the prevalence we find in rural China is comparable to that of family caregivers of adults with heart failure in Chengdu (31%, Hu et al., 2017), and considerably higher than that of family caregivers of Alzheimer's patients in Tianjin (6%, Liu et al., 2016).

A greater proportion of grandmother caregivers (34.7%) suffer from depression relative to mothers (18.9%). Of those depressed, it is notable that 66% of grandmothers scored within a moderate or higher severity of depression, compared to 50% of mothers, suggesting that grandmothers are also more likely to experience a greater severity of depressive symptoms. Our qualitative results support this, with 13 of the 16 depressed participants in our sample being grandmothers (81 vs. 19%). Other studies have consistently found that older, lower-income, less-educated women in rural areas are more likely to have depressive symptoms than younger women (Hou et al., 2015; Qiu et al., 2016). Interestingly, all five of the illiterate participants in our qualitative sample were grandmothers and depressed.

The prevalence of depression is worrisome, especially because we also find a positive association between caregiver depression and children's social-emotional and language delay in our multivariate analysis. Specifically, we show that caregiver depression is associated with a 0.53 SD worsening in children's social-emotional development, and a 1.56 point (0.12 SD) decrease in their language development. Around half of the children in our sample are significantly delayed in one or more of these two developmental areas. Developmental delays in children are well-known to present lasting consequences for their future. It is therefore imperative to understand the underlying features of caregiver depression in order to better prevent and treat depression in the future. Which caregivers are depressed? And more importantly, why are they depressed? We use a rich set of in-depth interviews to further investigate contributing factors to caregiver depression.

Our qualitative findings confirm all four of our original hypotheses, and also identify two additional factors contributing to depression. Confirming our hypotheses, we find that a lack of social support, the burden of childrearing, a lack of agency, and family conflict all contribute to depression. Beyond our hypotheses, we find that both poverty and the use of material wealth to measure self-worth are also important contributing factors. We discuss each of these six factors below.

First, we find that a lack of social support is a serious problem in rural villages. Social stigmas against seeking help, mental illness, and admitting difficulty at home exacerbate depression. These stigmas discourage women from opening up to others about troubles out of fear of judgment, mockery or gossip. Studies have consistently shown that individuals with higher levels of social support are less likely to be depressed, including in rural China (Grav et al., 2012; Hou et al., 2015). Greater social support as theorized in the Buffer Theory (defined as information that leads to one feeling esteemed, cared for, and loved) can protect people from both mental and physical illnesses, especially in challenging times (Cassel, 1976; Cobb, 1976).

Second, we find that depressed caregivers experience more distress from taking care of children than non-depressed caregivers. Our interviews suggest that the children of depressed caregivers are not more difficult to raise, but rather, that it is the caregiver who experiences childrearing to be more difficult. This could also be related to reduced levels of energy, a symptom of being depressed (Downey and Coyne, 1990). Numerous studies have shown that depressed caregivers reduce effortful interaction with their children, speaking less often and responding slower to their children than non-depressed mothers (Breznitz and Sherman, 1987; Bettes, 1988). Reducing effortful interaction can also lead to increased hostility and irritability toward their children, especially under stress (Cohn et al., 1986). Studies indicate that depressed mothers are more likely to unilaterally enforce obedience (resorting to harsh punishment, for example) as it requires less cognitive effort than negotiating with their child (Kuczynski, 1984). This may partially explain why more depressed caregivers in our sample admit to hitting their child when angry or frustrated than non-depressed caregivers. Caregivers who resort to harsh punishment are also less sensitive to their child's needs in general (Field et al., 2006; McLearn et al., 2006; Field, 2010).

Third, we find that conflict within the family—typically caused by a perceived failure of other family members to fill their social role—also contributes to depression. Depressed grandmothers often express their agitation with their daughter-in-law for not fulfilling a duty—sending money home, helping with housework, childcare, or birthing a son. However, we did not find this the other way: mothers seemed less distressed and worried about their mother-in-law. Many mothers stated they anticipated migrating to the cities for work within a few years and bringing their children with them. This may also account for the lack of young mothers in our sample who are distressed by a dominant mother-in-law. Young mothers, even those who live in uncomfortable situations, may understand that the situation is only temporary.

Fourth, a number of studies have supported the notion that depression is linked to the level of perceived control someone has over their life (Ross and Mirowsky, 1989, 1990; Kennedy, 2014). We find this to be true, though not quite in the way we expected. The opportunity available to younger women to migrate outside the village for work accounts for an important difference between grandmothers and mothers. Studies have shown an increasing number of migrants in China who are women (Connelly et al., 2012; Mu and de Brauw, 2015). This (relatively new) option to leave offers an outlet and form of escape for young mothers, who have the education and energy to leave the village. By contrast, it appears to be grandmothers who feel trapped in their villages. During our interviews, many depressed grandmothers associated their unhappiness with being unable to change their particular circumstances (“who would employ an illiterate grandmother?”).

An unanticipated fifth factor contributing to depression is poverty. This is consistent in both our quantitative and qualitative work. This is not a new finding, but confirms that poverty is also a strong risk factor for mental illnesses like depression (World Health Organization, 2012) in rural China. A review of 11 studies of mental disorders across six middle-income and low-income countries found that the majority concluded an association between poverty and mental illness (Patel and Kleinman, 2003).

In rural China, the role of poverty as a risk factor is compounded by a sixth factor: the centrality of material wealth to one's self-worth. Many caregivers were quick to associate “self-worth” with material assets, money, and jobs. If one had less money or fewer assets than their neighbor, it was perceived as a clear sign that they were literally “worth less.” As many caregivers note, simply seeing the wealth of their neighbors can stir feelings of having lived a meaningless life. For those who see their worth as directly related with their poverty, the situation is often exacerbated by the perception that others will always have more.

Our study contributes to depression research in rural China in two main ways. First, our quantitative dataset includes a rich set of early childhood development outcomes, allowing us to quantify the relationship between caregiver depression and child development. Second, our study is the first to combine both quantitative and qualitative data on the topic of caregiver depression, and the first to use an in-depth qualitative study to explore contributing factors to depression through open-ended interviews.

Our study faces several potential limitations that should be noted and addressed in future studies. Firstly, the data used are from a cross-sectional study, preventing us from making causal connections between caregiver depression and child development outcomes. Second, the study was conducted in only one northwestern province in China, limiting its external validity. Third, our presentation of prevalence of child development delay relies on international rather than Chinese norms and should therefore be interpreted with caution; no norms yet exist for the BSID-III for China specifically. Our correlational analyses, by contrast, rely on raw index scores rather than normed data, and are therefore unaffected by the lack of Chinese norms.

The use of Western-developed assessment tools to measure depression in Chinese communities can be questioned. A consistent finding among depression researchers in China is that distress in Chinese populations are expressed through “bodily complaints” rather than emotional feelings (Kleinman, 1985). This is evident in our sample as well (i.e., “my heart hurts,” or episodes of “not wanting to eat”). During our interviews we attempt to account for this potential shortcoming by allowing caregivers to explain their experiences freely and asking open-ended questions.

A 2007 ethnographic study of experiences of Chinese depression published in the Harvard Psychiatry Review found that in general, Euro-American psychiatry can in fact be applied to the Chinese experience (Lee et al., 2007). This is substantiated by recent work by Qiu et al. (2016) of women in rural Sichuan. They find that the majority of their sample's presentations of depression were compatible with DSM-based psychopathology (Qiu et al., 2016). They attribute this to the growing familiarity with Western understandings of depression in China among psychiatrists and patients themselves. However, given that we did find evidence of some of Kleinman's findings in our experiences with Chinese women, we would continue to recommend culturally sensitive approaches in the future.

## Conclusion

High depression rates among female caregivers of young children pose an important social and health problem in rural China. These women's depression both negatively influences the development of the young children they are raising, while also having a detrimental impact on the women's quality of life. We find that older caregivers, grandmothers, suffer more often, and more severely from depression than do younger mothers. Lack of social support, the burden of caregiving, and a perceived lack of agency are all important contributors, as are poverty, a perceived link between material wealth and self-worth, and within-family conflict.

Our findings show a serious lack of understanding of mental health issues among rural women, and suggest that rural communities could benefit greatly from an educational program concerning mental health and its influence on child development. The use of social media such as WeChat can be useful in spreading information and raising awareness; however, not everyone has access to mobile devices. Elderly people in particular do not always find their way on to social media. Future studies might explore delivery of educational programs both over social media and in rural communities themselves, with specific attention paid to elderly caregivers. Further research is also needed to better understand how depression may be affecting other types of primary caregivers in rural China, includingnon-mother non-grandmother family members who care for young children.

## Author Contributions

AY, MY, LS, AM, and SR developed the study concept and design. AY, MY, LS, AM, and JG oversaw fieldwork and data collection. AY, JG, MY, and LS analyzed the data. MY, LS and AM interpreted the results. AY, MY, LS, AM, and SR drafted the article. All authors were involved in critical revisions of the manuscript and approved the final version for publication.

### Conflict of Interest Statement

The authors declare that the research was conducted in the absence of any commercial or financial relationships that could be construed as a potential conflict of interest.

## Footnotes

^1^Principal Component Analysis (PCA) was constructed using the following variables: piped water, flush toilet, water heater, washing machine, computer, internet, refrigerator, air conditioning, motorcycle and car.
